# Bilateral Diffuse and Cluster Pigment Epithelial Detachment Associated with Diffuse Proliferative Glomerulonephritis

**DOI:** 10.1155/2018/5474696

**Published:** 2018-02-21

**Authors:** Heshmatollah Ghanbari, Alireza Dehghani, Mohsen Pourazizi

**Affiliations:** Isfahan Eye Research Center, Isfahan University of Medical Sciences, Isfahan, Iran

## Abstract

Retinal pigment epithelium detachment (PED) is an area of retinal pigment epithelium (RPE) elevation with minimal or no sensory retinal detachment resulting from the accumulation of sub-RPE fluid. There are many etiological factors that lead to the development of PED. PED may be observed as an isolated finding or in association with ocular and systemic conditions. In this case we report a 23-year-old male with bilateral cluster PED associated with diffuse proliferative glomerulonephritis (DPGN). The importance of current report is that development of PED and DPGN is more than a simple incidental event. Patients with DPGN should have regular fundus examinations, and follow-up should be conducted by an ophthalmologist who is aware of the possible presence of these diseases.

## 1. Background

A retinal pigment epithelium detachment (PED) is an area of retinal pigment epithelium (RPE) elevation with minimal or no sensory retinal detachment [1]. A PED presents usually as a focal lesion but occasionally it is evident as multiple lesions. PED can appear in a setting of serous detachment (serous PED) or choroidal neovascularization, also they can be detected as an isolated finding in fundus [2]. Although the exact etiology of PED is unknown, it is evident that any disorder that can destabilize the complex attachment of the basement membrane of the RPE and Bruch membrane will have an effect on PED [1, 3].

Possible causes for PED include inflammation, ischemia, and degenerative and idiopathic processes [1]. There are limited reports describing PED in association with ocular and systemic conditions associated with PED [2].

It is important to distinguish idiopathic PEDs from those occurring in association with other diseases. The purpose of this report was to illustrate the clinical and paraclinical features of a patient with bilateral diffuse and cluster PED in association of diffuse proliferative glomerulonephritis (DPGN). We also aimed to find the possible link between DPGN and PED by reviewing the pathophysiologic process reported in the literature.

## 2. Case Presentation

A 23-year-old white male with DPGN since 5 years was referred to our institute for a visual disturbance. His vision had gradually worsened in the last 2 years, but he did not consult any physician in this period. There was no history of photophobia, scotoma, metamorphopsia, or any other remarkable ocular symptoms.

The patient had no any history of ocular diseases or previous ocular surgery.

His family history was not noticeable for KC, other ocular, and renal problems.

Past medical history revealed previous renal problems. Five years earlier he had presented with massive proteinuria and had been diagnosed with DPGN, which was confirmed using renal biopsy and treated with Tacrolimus, Cyclophosphamide (CTX), and prednisolone. At the time he presented before us, he had proteinuria and persistent low-level C3 despite the cytotoxic therapy with CTX, Mycophenolate Mofetil, and prednisolone.

A complete medical evaluation including a comprehensive ocular and systemic history, complete blood count, blood chemistry, erythrocyte sedimentation rate, antinuclear antibodies and serum autoimmune antibody, anti-GBM antibodies, chest radiographs, purified protein derivative test, and consultation with a rheumatologist failed to find any systemic etiology, including infectious systemic disorders, systemic inflammatory diseases (SLE, anti-GBM antibodies), systemic vascular occlusion, and Alport disease, at the time of the diagnosis of the renal problem before the initiation of corticosteroid and immunosuppressive therapy.

On ophthalmologic examination, his uncorrected visual acuity (UCVA) was 8/40 in right eye and 10/40 in left eye and best-corrected visual acuity (BCVA) was 10/40 in right eye (OD; −5.25/−6.50 × 30) and 20/40 in left eye (OS; −2.00/−4.00 × 95). There was no relative afferent papillary defect (RAPD).

On anterior segment examination, the corneas were clear bilaterally but the examination revealed a paracentral cone in both eyes.

**Figure 1:**
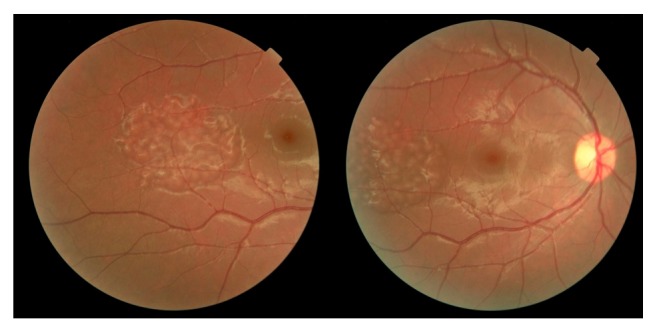
Fundus photograph. Fundus photograph of right eye showing a locally elevated region with well-defined margins and a diffuse cluster pattern on peripheral fundus corresponding to the diffuse and cluster pigment epithelial detachments.

**Figure 2:**
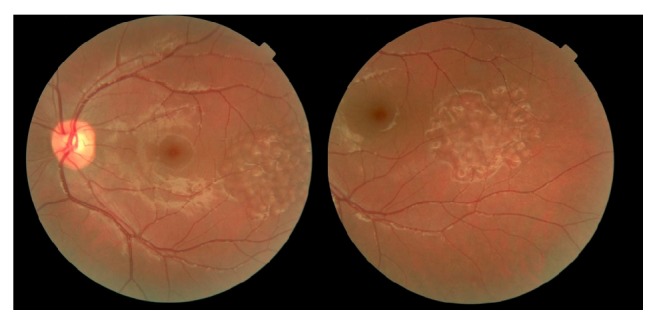
Fundus photograph. Fundus photograph of left eye showing a locally elevated region with well-defined margins and a diffuse cluster pattern on peripheral fundus corresponding to the diffuse pigment epithelial detachments.

Fundus examination revealed multiple and diffuse pale elevated area with well-defined margins and same sizes in diffuse and cluster pattern in the temporal of macula but he had no maculopathy (Figures 1 and 2). Optic discs were normal in both eyes; there was no evidence of basal drusen, hemorrhages, hard exudates, choroidal neovascularization, posterior vasculitis, or vascular occlusion.

**Figure 3:**
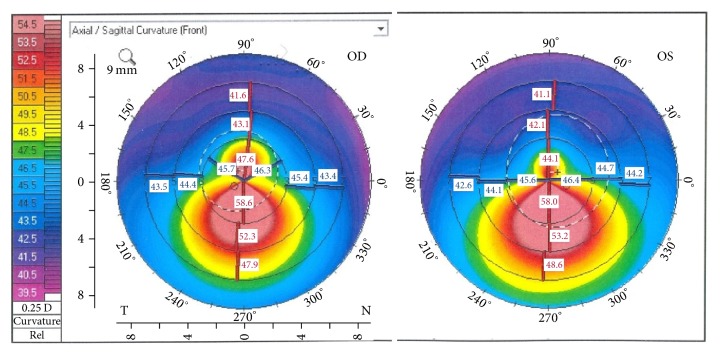
OCULUS Pentacam®. Bilateral inferior corneal steepening, indicative of keratoconus.

Corneal topography (anterior surface) demonstrated bilateral KC that was worse on the right eye than the left. Keratometric findings were RE (K1: 47.1 and K2: 53.0) and LE (K1: 46.1 and K2: 50.5). Pachymetry showed thinned corneas corneas (OD 478 *μ*m centrally, thinnest 467 *μ*m; OS 491 *μ*m centrally, thinnest 480 *μ*m) (Figure 3).

**Figure 4:**
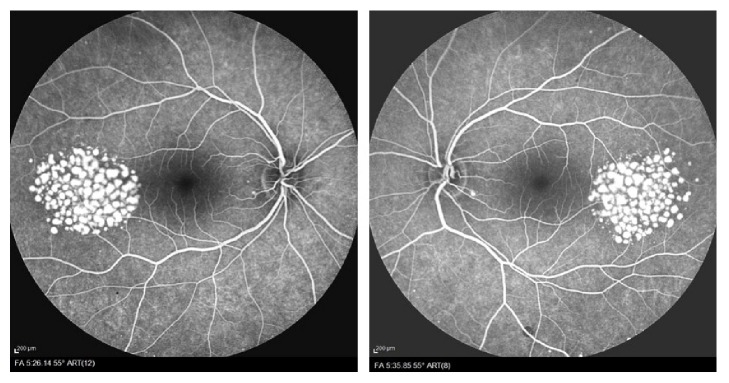
Fluorescein angiograms. Fluorescein angiograms of both eyes, showing bilateral hyperfluorescence of multiple small, oval to round lesions, with increasing of intensity at later phases without any leakage.

Fluorescein Angiofluoresceingraphy (FFA) (Heidelberg Engineering, Germany) showed diffuse and cluster area of hyperfluorescence (fluorescence pooling) in early times but no findings of leakage, which corresponded with the pale areas (Figure 4).

**Figure 5:**
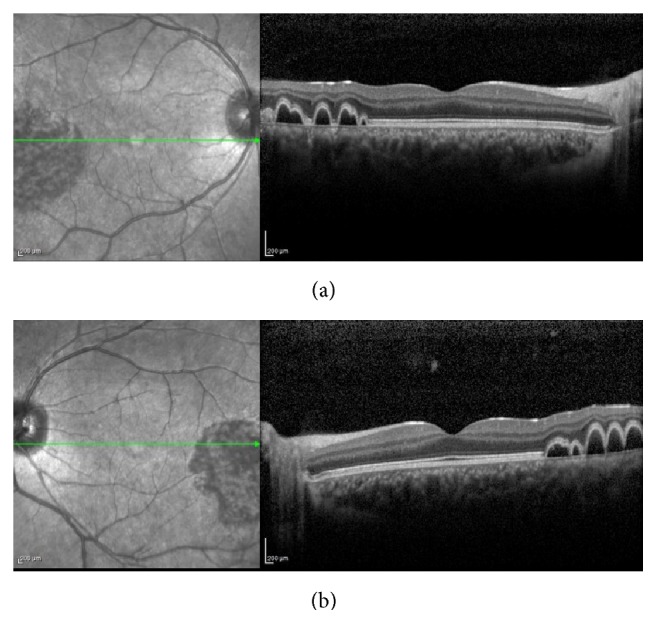
Optical coherence tomography (OCT). OCT of right (a) and left (b) eyes, showing bilateral multiple small-size dome-shaped retinal pigment epithelial detachments.

Optical coherence tomography (OCT) (Heidelberg Engineering, Germany) illustrated bilateral, multiple small-size dome-shaped lesions, compatible with PED (Figure 5).

Based on FFA, OCT, and clinical findings, the diagnosis of bilateral diffuse and cluster PED and KC was made.

We did not treat our patient for PED. To manage KC, the patient attempted wearing contact lenses but he did not tolerate them. Therefore, a collagen cross-linking (CCL) was planned for both eyes. However, there are surgical interventions options for the future should his condition progress to an unacceptable level. The patient was advised to return for regular follow-up. The PEDs did not change during of the 10 months of follow-up

## 3. Discussion

We described a case of bilateral diffuse and cluster pigment epithelial detachment associated with DPGN. The point that made this case impressive was its bilateral diffuses and cluster distributions in association with DPGN.

The association of RPE alterations with one type of glomerulonephritis was first reported in 1989 [4].

RPE detachment of retina is a rare clinical presentation in the course of a systemic disease. PED is usually observed as an isolated finding, although, in rare cases, they may also occur in association with other systemic diseases, including renal diseases [2].

Several hypotheses have been proposed for detachment of RPE resulting from the disintegration of the RPE union with the collagen layer of Bruch's membrane [1, 5], but the most important reason is alternation in choroidal vascular permeability [3].

A review of the literature reveals several reports describing PED in association with ocular and systemic conditions [2]. Renal causes of PED are thought to originate in the deposition of antibodies on the basement membranes of the RPE and may lead to breakdown and detachment of RPE [2]. PED may also occur in association with serous retinal detachments in hemodialytic patients [6].

Other renal disorders, such as IgA nephropathy, also have been reported in association with RPE detachment of the retina [7].

PED also may occur in the setting of type II membranoproliferative glomerulonephritis (MPGN) with or without serous retinal detachment [8].

It is important to identify the specific nature of detachment of the RPE and to establish an accurate diagnosis [1, 5].

The possible association between KC and PED in patients affected by glomerulonephritis is an important clinical stimulus for paying high attention to the specific characteristics of both anterior and posterior segments, during the ophthalmological examination of this type of patients.

Coexistence of these two distinct disorders in our patient mostly reflects the possible presence of collagen or cellar matrix component disturbance or antibody formation against the eyes and kidneys.

The basement membrane of the RPE joins the Bruch membrane in the hemidesmosome regions, which contains laminin filaments, collagen types IV and V, and proteoglycans [1].

The Bowman layer is composed of type IV collagen, laminin, and other proteins. Beneath the acellular Bowman layer, the corneal stroma is composed of an extracellular matrix formed by collagens and proteoglycans. Type I and type V fibrillar collagens are intertwined with filaments of type VI collagen [3, 9]. Given this situation of the existence of similar components (e.g., collagens) in the pathophysiology of PED and DPGN, our report might suggest a link between these diseases. However, the exact association of such linkages would be better clarified in more immunohistopathological and medical genetics studies.

Since RPE changes have not been observed in all type of GN, the association between renal disease and RPE alternations may help nephrologists make the diagnosis in those presenting with unclassified GN. We thereby suggest the possible linkages between PED and DPGN.

## 4. Conclusion

The development of PED and KC in individuals with DPGN could be more than a simple incidental event and further study will be performed to confirm this hypothesis. Patients with DPGN should have regular fundus examinations, and follow-up should be conducted by an ophthalmologist who is aware of the possible presence of these diseases.
